# Bonfils intubation fibrescope: use in simulation-based intubation training for medical students in comparison to MacIntosh laryngoscope

**DOI:** 10.1186/s13104-016-1937-2

**Published:** 2016-02-27

**Authors:** Tobias Limbach, Thomas Ott, Jan Griesinger, Antje Jahn-Eimermacher, Tim Piepho

**Affiliations:** Department of Anaesthesiology, University Medical Centre of the Johannes Gutenberg University Mainz, Langenbeckstr 1, Mainz, 55131 Germany; Institute of Medical Biostatistics, Epidemiology, and Informatics, University Medical Centre, Johannes Gutenberg University Mainz, Langenbeckstr 1, Mainz, 55131 Germany

**Keywords:** Airway management, Intubation, Bonfils, Simulation, Students’ training

## Abstract

**Background:**

A variety of instruments are used to perform airway management by tracheal intubation. In this study, we compared the MacIntosh balde (MB) laryngoscope with the Bonfils intubation fibrescope as intubation techniques. The aim of this study was to identify the technique (MB or Bonfils) that would allow students in their last year of medical school to perform tracheal intubation faster and with a higher success probability. Data were collected from 150 participants using an airway simulator [‘Laerdal Airway Management Trainer’ (Laerdal Medical AS, Stavanger, Norway)]. The participants were randomly assigned to a sequence of techniques to use. Four consecutive intubation ‘trials’ were performed with each technique. These trials were evaluated for differences in the following categories: the ‘time to successful ventilation‘, ‘success probability’ within 90 s,’time to visualisation’ of the vocal cords (glottis), and ‘quality of visualisation’ according to the Cormack and Lehane score (C&L, grade 1–4). The primary endpoint was the ‘time to successful ventilation‘in the fourth and final trial.

**Results:**

There was no statistically significant difference in the ‘time to successful ventilation’ between the two techniques in trial 4 (‘time to successful ventilation’: median: MB: 16 s, Bonfils: 14 s, p = 0.244). However, the ‘success probability’ within 90 s was higher when using a Macintosh blade than when using a Bonfils (95 vs. 87 %). The glottis could be better visualised when using a Bonfils (C&L score of 1 (best view): MB: 41 %, Bonfils: 93 %), but visualisation was achieved more rapidly when using a Macintosh blade (median: ‘time to visualisation’: MB: 6 s, Bonfils: 8 s, p = 0.003).

**Conclusions:**

The time to ventilation using the MacIntosh blade and Bonfils mainly did to differ, however success probabilities and time to visualisation primary favoured the MacIntosh blade as intubation technique, although the Bonfils seem to have a steeper learning curve. The Bonfils is still a promising intubation technique and might be easier to learn as the MB, at least in a manikin.

**Electronic supplementary material:**

The online version of this article (doi:10.1186/s13104-016-1937-2) contains supplementary material, which is available to authorized users.

## Background

The Bonfils rigid fibrescope, an airway instrument for tracheal intubation, was first described in 1983 [1], and it has been evaluated for elective [2, 3] and emergency intubation [4, 5]. It has shown advantages in managing difficult airway situations under both real [6–10] and simulated conditions [11–13] when applied by experienced providers. The Bonfils fibrescope has also been evaluated in simulation-based studies, revealing acceptable ease of use by novice anesthesiologists [14], skilled anesthesiologists and physicians of other disciplines [15, 16]. Tracheal intubation is an advanced airway management skill that should be performed by experienced providers explicitly [17–19]. However, in clinical reality, intubation is performed by inexperienced providers in a relatively large number of cases [20, 21]. Notably, unsuccessful intubation attempts have been associated with a higher rate of severe complications in emergency patients [22].

Medical students present a high level and broad field of theoretical knowledge but lack practical experience and therefore, they should be trained in using supraglottic airway instruments [19]. However, particularly in emergency departments and intensive care units, tracheal intubation must be mastered by novices without the recommended experience. Therefore, it is inevitable that tracheal intubation and alternative instruments for achieving proper oxygenation and ventilation are mandatory training subjects included in medical schools’ curricula [23, 24]. A classical laryngoscope with a Macintosh blade (MB) is the instrument that is most frequently used for tracheal intubation; thus, it is included in our curriculum as a mandatory training subject. Several learning curves have been described for the MB [18, 25], and success rates and speed of intubation mainly depend on the experience of the particular user [26].

Most medical students do not have experience with using an MB [24], but they may be more accepting of the use of alternative intubation techniques than experienced users. Our approach was to compare the use of the Bonfils fibrescope as an older, infrequently used instrument with that of the MB by students to obtain unbiased data due to the students’ impartialities towards unknown techniques.

Our hypothesis was that the time students need to perform simulated intubation after a training period of three trials depends on the technique (Bonfils fibrescope or MB).

Our primary endpoint was the difference in the ‘time to successful ventilation’ with a tracheal tube achieved using an MB and Bonfils fibrescope in the last of four intubation trials. The secondary endpoints included the differences in the ‘success probability’, ‘time to visualisation’ of the glottis, ‘quality of visualisation’ according to the Cormack and Lehane (C&L) score and appearance of simulated tooth damage.

## Methods

After obtaining approval from the institutional review board (Data Protection Commission, University Medical Centre of the Johannes Gutenberg-University Mainz, Confirmation of 12. February 2012, Dr. I. Reinisch), we conducted this study as part of mandatory clinical skills training included in our medical school curriculum at the start of the final year of medical school, referred to as ‘MINERVA’ (Mainz Initiative for Intensive Novelized Excellence Trending of Versatile Apprenticeship). We obtained written informed consent from the participants before data collection, including consent for participation and permission to publish the data anonymously. Refusal of participation in the study had no consequences on the workshop curriculum.

### Bonfils intubation fibrescope

The retromolar Bonfils intubation fibrescope [1] (Karl Storz GmbH, Tuttlingen, Germany) has been available for purchase since the mid-1980s. The proximal aspect consists of an eyepiece, an adapter for a light source and a handle that leads to a 40 cm long, 5 mm thick metal stylet. The distal tip of the stylet has a 40° ventral bend and contains the optic element of the eyepiece and the light fibre. To conduct tracheal intubation, a conventional tube is pulled over the stylet so that the optic element offers an open view. It is recommended that the retropharyngeal space is widened by the jaw thrust and that the Bonfils fibrescope is introduced strictly using a retromolar approach. Then, the tube is pushed forward through the vocal cord. The Bonfils is not advanced through the vocal cord at any time [14]. Essential advantages over direct laryngoscopy with an MB are that only minimal mouth opening and movement of the cervical spine are required during the intubation process [3].

### Simulator

This study was conducted using a ‘Laerdal Airway Management Trainer’ (Laerdal Medical AS, Stavanger, Norway). This simulator offers two possible positions for tube insertion to an adequate depth: tracheal or oesophageal. When the tube is placed in the trachea and the cuff is blocked with 10 mm of air, two freely suspended lungs inflate to simulate a ventilation attempt. When the tube is placed in the oesophagus, a stomach balloon is inflated.

### Participants

One hundred fifty students in their last year of medical school were invited to participate in the study, and we obtained written informed consent from all of them. The sample included 79 (53 %) female and 71 (47 %) male participants, with a mean age of 26.7 years. All of the participants had one semester of resuscitation training during the 1st year of medical school, including mask ventilation and supraglottic airway management. The students also attended an anaesthesia lecture and acquired 4 days of operating room experience in anaesthesia during their 2nd year. The students were not required to perform tracheal intubation on patients, but they did perform mask ventilation under supervision. Further, they obtained 1 h of practical training with simulators for mask ventilation, supraglottic airway management and tracheal intubation using an MB and several alternative devices for tracheal intubation, but not a Bonfils fibrescope, during their 4th year. A total of 69 students (46 %) had not performed intubation on any patient until participating in this study, 54 (36 %) had performed up to ten intubations, 26 (17 %) had performed upto 50 intubations, and one had performed up to 100 intubations. We analysed all of the students to reliably detect intraindividual differences between the two intubation devices. For analysis of subgroups, the participants were divided into two groups: female and male.

For the workshop, the students were randomly assigned to groups of six according to the first letter of their surname.

### Data collection

Before data collection, the participants were randomly assigned a sequence of techniques to use. The C&L scoring system was explained, and a poster illustrating it was presented visibly above the airway simulator. The participants were not allowed to train on the instruments before the study. TL and JG taught the participants how to perform intubation, beginning with alternating usage of the conventional laryngoscope with an MB and a Bonfils fibrescope, following the ‘four-step approach‘described by Peyton [27, 28] for each technique. First, intubation was demonstrated by one of the instructors in real time with assistance from another instructor. Second, intubation was demonstrated again with a detailed explanation. Additionally a part model of the larynx was used for this purpose. Third, the participants had to explain the intubation procedure to an instructor, who simultaneously performed the procedure according to the participant’s explanation. Fourth, each participant had to perform tracheal intubation once alone. Then, training for the other technique proceeded in the same manner.

Thereafter, each participant had to attempt intubation four times (i.e. four intubation trials) with each technique.

A trial was conducted as follows: the participants held the particular instrument in their hand and stood in front of the simulator, and a stop watch was started as soon as the instrument was passed through the lips of the simulator. The following data were recorded:

The ‘time to visualisation’, ‘time to successful ventilation’, ‘success’ of the intubation attempt’, ‘visualisation of the glottis’ and ‘tooth damage’. Precise definitions of the data collected were as follows:

1. ‘Time to visualisation’: the time from the start of the attempt until the participants announced that they could visualise the glottis.
2. ‘Time to successful ventilation’: the time from the start of the attempt until the first sign of successful ventilation (inflation of the lung) was detected.
3. ‘Success’: an intubation attempt ending with inflation of the lungs using a conventional self-inflating ventilation bag within 90 s. If only one lung was inflated, the attempt was also considered successful, but the participant was advised to adjust the depth of the tube correctly afterwards. If the lungs had not inflated or if they had inflated after a duration of more than 90 s, then the attempt was considered unsuccessful.
4. ‘Visualisation of the glottis,’ according C&L: after each ventilation attempt, the participant had to state the achieved C&L score [29]. This score was given by the participant and was not revaluated by an instructor.
5. ‘Tooth damage’: the upper row of teeth generates a ‘clicking’ sound if the teeth are pushed too hard in the cranial direction. If a clicking sound occurred during an intubation attempt, the event was considered to have caused tooth damage.

The participants conducted four consecutive trials with each technique. Then, they had to state which technique that they preferred to use for tracheal intubation. Finally, their previous experience with tracheal intubation was recorded.

### Study outcomes

The primary endpoint was the ‘time to successful ventilation’ in the fourth and final trial. This time point was chosen so that all of the participants performed at least three ventilation attempts with the equipment.

The secondary endpoints were as follows: the ‘time to successful ventilation’ in trials 1 through 3, ‘success probability’, ‘time to visualisation’, ‘quality of visualisation’ (C&L), and incidence of tooth damage in each trial and technique preference (MB or Bonfils). Additionally, we conducted subgroup analysis to assess the influence of gender on ‘time to ventilation’ and ‘success probability’.

### Statistical analysis

Our main objectives were the intraindividual differences in the applicability aspects of the two techniques. Thus, we assessed the data per subject as tied observations. As primary analysis, the time to successful ventilation in trial four is compared between the techniques by applying the stratified Cox proportional hazards model, with the technique as a covariate and the participant as a stratum. The two-sided Wald test is calculated at a significance level of 5 % to test the null hypothesis, that subjects need the same time for successful ventilation when using the MB or the Bonfils technique. Kaplan–Meier plots are provided to visualize results. The same analyses are performed as secondary analyses for trial 1–3 and for visualization over time. Furthermore, for each trial, the absolute and relative frequencies of successful ventilation within 90 s were determined, and the techniques were compared using the McNemar test for intraindividual comparisons. The C&L score and tooth damage were described as absolute and relative frequencies per trial. For all secondary endpoints, p values are presented for descriptive purposes, without adjustments for multiple testing. Data analysis was performed using SPSS version 22 (IBM, Ehningen, Germany) and R open-source statistical environment, version 3.1.0 (2014-04-10) [30].

## Results

One hundred fifty participants were included in the study. For one participant data on time to successful ventilation and time to visualisation when using the MB technique is missing for all trials.

### Primary endpoint

**Fig. 1:**
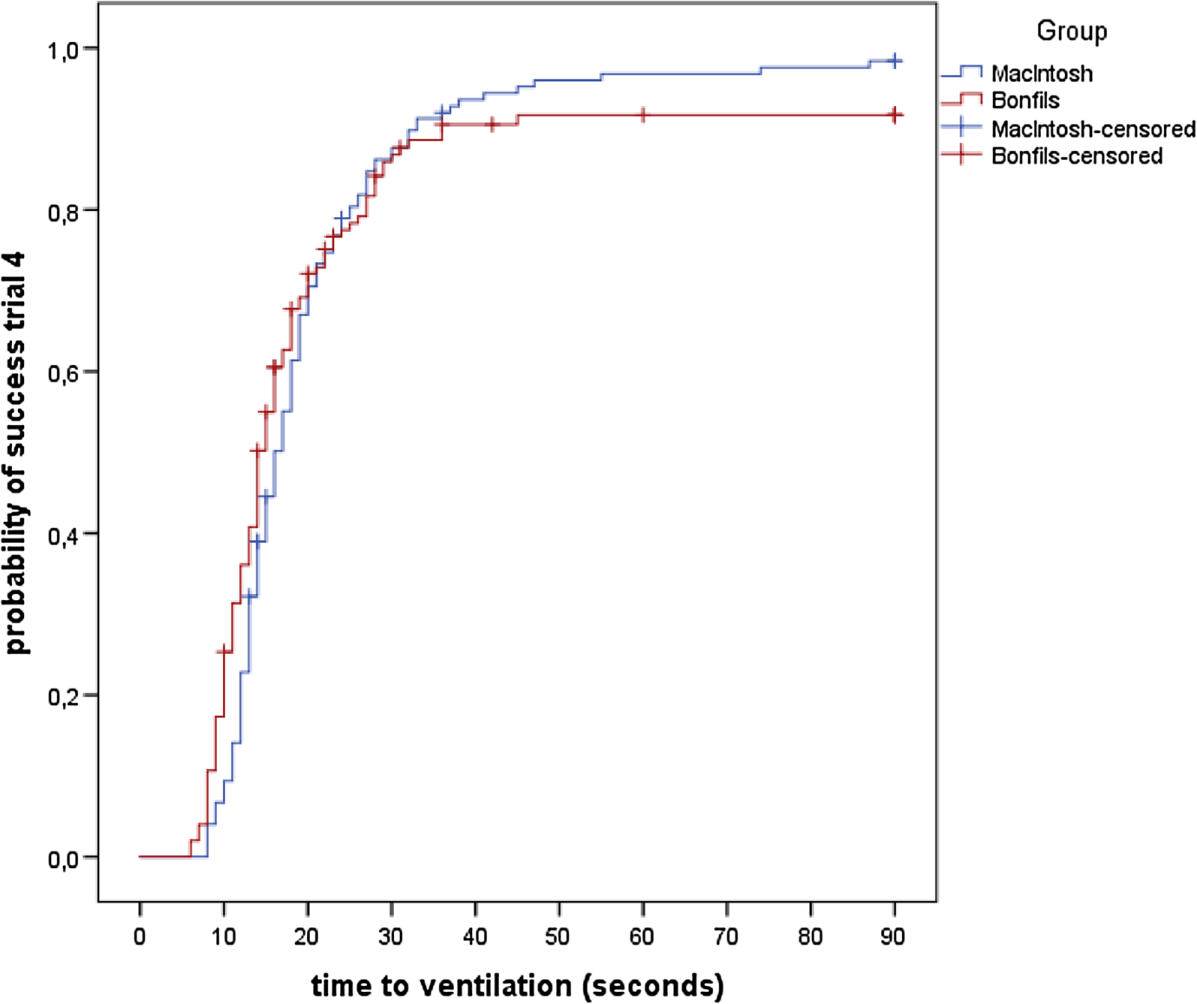
‘Probability of success’ in trial 4. ‘Probability of success’ over time (*sec*) in trial 4 with the Macintosh blade (*blue line*) and Bonfils intubation fibrescope (*red line*); unsuccessful attempts and those taking longer than 90 s were censored (*cross on the lines*)

There was no statistically significant difference in the ‘time to successful ventilation’ between the techniques for the fourth and final trial (median: MB: 16 s, Bonfils: 14 s, p = 0.244) (Table 1; Fig. 1).

**Table 1:** Median ‘time to successful ventilation’ and ‘time to visualisation’ (in sec) in trials 1 through 4

Trial	1		2		3		4	
Technique	MB	Bonfils	MB	Bonfils	MB	Bonfils	MB	Bonfils
Time to successful ventilation								
Median	26	32	20	23	17	17	16	14
95 % CI	23–29	28–36	19–21	20–26	16–18	15–19	15–18	13–15
n (censored)	10	40	1	37	4	24	8	21
p	0.007		0.238		0.458		0.244	
Time to visualization								
Median	12	21	8	14	6	9	6	8
95 % CI	10–14	16–26	7–9	11–17	5–7	8–10	5–7	7–9
n (censored)	10	40	1	37	4	24	8	21
p	<0.001		<0.001		0.001		0.003	

The primary endpoint was ‘time to successful ventilation’ trial 4. Time points: consecutive trials 1, 2, 3, and 4; the median of the ‘time to successful ventilation’ and ‘time to visualisation’ for each technique: *MB* Macintosh blade, *Bonfils* Bonfils intubation fibrescope, CI confidence interval, and n (censored): the number of unsuccessful attempts and those taking longer than 90 s; p: Wald p value from the stratified Cox model testing for the difference between the techniques in each trial [missing data (n = 1)] for participant under the MB in all trials

### Secondary endpoints

In trial 1, the participants took longer to achieve successful intubation using a Bonfils, and for trials 2–4, the ‘times to successful ventilation’ were similar for both techniques.

There was a tendency towards a higher ‘success probability’ for the subjects using an MB compared with those using a Bonfils (Table 2). In trial 1, 139 (93 %) out of 150 participants performed a successful simulated intubation with an MB, and 109 (73 %) were successful with a Bonfils. In trial 4, 141 (95 %) of the participants performed a successful intubation with an MB, and 129 (87 %) achieved success with a Bonfils.

**Table 2:** ‘Success probabilities’

Trial:		1		2		3		4	
Technique	Bonfils								
MB	Success	Yes	No	Yes	No	Yes	No	Yes	No
	Yes	103 (69 %)	36 (24 %)	111 (75 %)	37 (25 %)	123 (83 %)	22 (15 %)	121 (81 %)	20 (13 %)
	No	6 (4 %)	4 (3 %)	1 (1 %)	0 (0 %)	2 (1 %)	2 (1 %)	8 (5 %)	0 (0 %)
	p	<0.01		<0.01		<0.01		0.036	

Time points: consecutive trials 1, 2, 3, and 4; technique: *MB* Macintosh blade, *Bonfils* Bonfils intubation fibrescope, the numbers (%) of participants with successful (yes) and unsuccessful (no) intubation attempts for each trial; the p values (McNemar test) for comparison of the success probabilities between the techniques in each trial. [missing value (n = 1)]

The participants using an MB had a shorter ‘time to visualisation’ of the vocal cords (Table 1), whereas the ‘quality of visualisation’ according to the C&L score was better when using a Bonfils (Table 3) across all four trials.

**Table 3:** Visualisation of the glottis according to the Cormack and Lehane (C&L) score

Trial	1		2		3		4	
Technique	MB (%)	Bonfils (%)	MB (%)	Bonfils (%)	MB (%)	Bonfils (%)	MB (%)	Bonfils (%)
C&L 1	29	84	34	89	43	91	41	93
C&L 2	63	14	60	10	51	7	52	6
C&L 3	6	1	7	1	7	1	5	0
C&L 4	2	1	0	1	0	1	1	1

Time points: consecutive trials 1, 2, 3, and 4; C&L scores of 1 to 4 for each technique: MB (Macintosh blade) and Bonfils (Bonfils intubation fibrescope)

Application of an MB resulted in a greater probability of tooth damage across the four trials compared with that of a Bonfils. In trial 4, tooth damage occurred during 13 % of all intubation attempts for the students using an MB, but this probability was only 2 % for those using a Bonfils.

A total of 51 (34 %) of the participants preferred using an MB, whereas 99 (66 %) preferred using a Bonfils.

The participants then rated both techniques on a scale, ranging from 1 (best) to 6 (worst), resulting in mean ratings of 1.9 for the MB and 2.2 for the Bonfils.

Concerning subgroup analyses, when using a Bonfils, female participants tend to achieve ventilation slower in most trials compared to male participants (Additional file 1) with lower success probabilities (Additional file 2). When using the MB, time to ventilation and success probabilities were similar between female and male participants.

## Discussion

This study identified no significant difference in the ‘time to successful ventilation’ between the MB and Bonfils in the last of the four consecutive intubation trials performed on a simulator.

Exploratory secondary analyses revealed a longer time to successful ventilation for Bonfils exclusively in the first trial but not for the second and third. Higher success probabilities were achieved with the MB than with the Bonfils. But success probabilities increased ca. 2 % for MB and ca. 14 % for Bonfils throughout the trials. The glottis was visualised more rapidly with the MB. The ‘quality of visualisation’ according to the C&L score was better with the Bonfils, and less simulated tooth damage occurred when using the Bonfils.

These results indicate that the participants who performed successful simulated intubation with a Bonfils yielded a better ‘quality of visualisation’ and the time needed for successful ventilation was similar between the Bonfils and MB.

Subgroup analysis indicated that both genders perform similar when using the MB. However, when using the Bonfils, female participants tend to show a slightly longer time to ventilation in 3 of 4 trials and a lower success probability throughout all trials than male participants.

To our knowledge, this is the first simulator-based study to explore inclusion of the Bonfils fibrescope in medical students’ training.

The results of our study are congruent to previous publications. Application of a Bonfils by novice anaesthesiologists with no prior experience in using it resulted in a success probability of 82 % and a time to successful ventilation of less than 30 s for the first attempt, as the airway of the simulator was configured normally. Like in our study, ventilation was achieved faster and was more often successful for the participants using the MB than for those using a Bonfils [14]. Another study evaluating the use of a Bonfils by novices (operating department practitioners and paramedics) revealed that the time to visualisation of the vocal cord like in our study was approximately 10 s and that the time to intubation was approximately 20-30 s for the first attempt [31]. In a study of anaesthesia practitioners (nurses and physicians), the time to intubation was found to be 22 ± 12 s, but the success rate was approximately 70 % for the first attempt, and are therefore similar to our results. C&L scores of 1 and 2 were achieved by 81 % of the participants [32]. Trainees with less than 3 years of anaesthesia experience have been reported to require 26 ± 28 s to intubate a simulator using a Bonfils, whereas skilled anaesthesiologists require 51 ± 50 s. The authors suggested that this finding was due to the increased exposure of younger physicians to video games or musical instruments compared with older physicians, which may have resulted in a better eye-hand-brain coordination [15]. However the exposure to video games and musical instruments might not explain sufficiently the gender differences seen in our study. Since the gender issue is not a primary endpoint of our study, the discussion of these differences has to be made with great caution. The intubation technique Bonfils might be easier for male than for female participants within four trials of attempts. However, reasons for these findings are not derivable with our study design and are not to be generalised for intubation issues.

Reports have stated that experience with performing 35 to 200 intubations in patients is necessary to achieve a success rate of at least 80 % by direct laryngoscopy with an MB [18, 25]. The authors doubt that a success rate of 80 % is sufficient for emergency situations. An association between the number of intubation attempts before successful intubation of emergency patients and severe complications has been reported [33]. In this context, the skill of the provider, especially in difficult airway situations, is crucial [17]. Thus, airway management is an important issue that must be included in medical schools’ curricula, and students must be trained in bag-mask ventilation [34], application of alternative airway devices [19] and tracheal intubation [35]. Deficits in airway management training are evident in a survey of over 600 junior doctors in Germany. Of the five main areas of competence deficit, lack of training in tracheal intubation was conceded by 43.5 % of all respondents [24]. According to the recommendations of the German Society of Anaesthesiology and Intensive Medicine, a physician should conduct at least 100 tracheal intubations in patients before taking responsibility in emergency medicine [36]. In contrast, other studies have reported that 10–25 intubations are necessary to gain enough experience to safely perform intubations on patients using a Bonfils [2, 15].

In a study by Herbstreit et al. participants with no experience in airway management, a success rate of 51 % for simulated direct laryngoscopy was observed in the first trial using an MB. After the participants took part in a defined training phase to perform this technique on patients in a clinical setting, the success rate of a single simulated intubation attempt was 71 % [37]. These findings indicate that the participants of our study, presenting success probabilities of over 65 % in the first trial using the MB, gained some favourable airway experience through their medical school curriculum concerning the MB. As well that there might be a training effect of the simulator. However, our participants attained a success probability of 87 % after only 4 trials on a simulator using a Bonfils. This finding again indicates that the learning curve of the Bonfils might be steeper than that of the MB when training on a simulator and may be even on patients. This factor might positively influence the value of the Bonfils as an alternative intubation technique.

Data collected using simulators always bear limitations concerning the translation to medical reality. A duration of 14 s until successful intubation and ventilation using a Bonfils after only four trials is obviously lower than that published for application in patients [38]. This study was conducted in an environment that was free from stress, with good lighting conditions, a comfortable working height and limited focus of the participant only on intubation and ventilation. This situation only mirrors the environment of emergency medicine to a very minor degree. The airway of the simulator considerably differs from that of real patients, for example, there is no mucus or saliva affecting sight and no coughing or deglutition occurs. The tongue of the simulator is stiffer than that of most patients, and the retropharyngeal space is wider, allowing for easier visualisation by the provider [39]. Further, concerning evaluation of study data, the C&L Score was reported by the participants and was not recheck by an instructor.

Sixty-six per cent of the participants preferred using a Bonfils, and only 34 % preferred using an MB. The reasons for this choice might include the less labour-intensive technique of the Bonfils, the good visualisation of the glottis and curiosity of the instrument, which was new to our participants. Other studies of providers with experience in airway management have shown stronger inclination towards an MB for patients with a normal airway [14, 31]. Inexperienced participants seem to be less biased against new techniques.

Considering the high frequency of intubations performed by inexperienced physicians and the success probabilities of the present and previous studies, all physicians working in any field of emergency medicine must be trained in tracheal intubation using an MB. Additionally, medical students must be trained on simulators before graduating from medical school. However, mask-bag ventilation is the primary skill applied for the oxygenation and ventilation of emergency patients [19, 40]. Particularly in medical school curriculum, training for mask-bag ventilation and the use of supraglottic airway devices must be performed repeatedly before focusing on tracheal intubation. Nevertheless, tracheal intubation must be included in all medical schools’ curricula.

Commonly, the application of the Bonfils is considered more difficult than that of the MB. This discrepancy might be due to the fact that the Bonfils is not ubiquitously present in the clinical setting; therefore, it is not addressed in medical school curriculum.

## Conclusions

The ‘time to successful ventilation’ of the MacIntosh blade laryngoscope and Bonfils were not found to differ in this manikin based study after four trials of intubation attempts. The success probabilities and ‘time to visualisation’ of the vocal cord at first glance favoured the MacIntosh blade as intubation technique; however at second glance the Bonfils seems to have a steeper learning curve concerning these items. Against this background the Bonfils is a promising intubation technique and might be easier to learn than the MB, at least in a manikin. Students’ training currently focus on the MB due to its clinical availability and broad application, however the Bonfils may have the potential to outperform the MB even in inexperienced providers.

## Availability of data and materials

The datasets and a description of the dataset supporting the conclusions of this article are available at LabArchives, LLC.: https://mynotebook.labarchives.com/doi/MTI3OTE0Ljh8OTgzOTYvOTgzOTYvTm90ZWJvb2svMTEwMDc4OTEwM3wzMjQ3MDYuOA==/10.6070/H4V98635 or via doi: 10.6070/H4V98635. For reasons of anonymity we published the data without the categories ‘age’.

## Additional files

10.1186/s13104-016-1937-2 Median ‘time to successful ventilation’ (in sec) in trials 1–4 for female and male participants.
10.1186/s13104-016-1937-2 ‘Success probabilities’ of the female and male participants.
