# Supplementary material for: Bonfils intubation fibrescope: use in simulation-based intubation training for medical students in comparison to MacIntosh laryngoscope
Source: BMC Res Notes. 2016 Feb 27;9:127. doi: 10.1186/s13104-016-1937-2 (PMC4769496; doi:10.1186/s13104-016-1937-2)
Supplement: Supplementary file 1 — 10.1186/s13104-016-1937-2 Median ‘time to successful ventilation’ (in sec) in trials 1–4 for female and male participants. [file 13104_2016_1937_MOESM1_ESM.docx]

Additional file 1: Median ‘time to successful ventilation’ (in sec) in trials 1 to 4 for female and male participants

|  |  |  | |  | |  | |  | |  | |  |  |  |  |  |  |
| --- | --- | --- | --- | --- | --- | --- | --- | --- | --- | --- | --- | --- | --- | --- | --- | --- | --- |
| MB trial | | | | 1 | | | | 2 | | | | 3 | | | | 4 | |
| gender | | | | female | | male | | female | | male | | female | | | male | female | male |
| median | | | | 27 | | 24 | | 20 | | 19 | | 18 | | | 16 | 17 | 16 |
| 95% CI | | | | 22-32 | | 21-27 | | 19-21 | | 18-21 | | 16-20 | | | 15-17 | 15-19 | 14-18 |
| n (censored) | | | | 4 | | 6 | | 1 | | 0 | | 2 | | | 2 | 5 | 3 |
| p | | | | 0.979 | | | | 0.275 | | | | 0.028 | | | | 0.113 | |
|  |  |  | |  | |  | |  | |  | |  |  |  |  |  |  |
| Bonfils trial | | | | 1 | | | | 2 | | | | 3 | | | | 4 | |
| gender | | | | female | | male | | female | | male | | female | | | male | female | male |
| median | | | | 40 | | 28 | | 24 | | 20 | | 21 | | | 15 | 16 | 13 |
| 95% CI | | | | 32-48 | | 24-32 | | 20-28 | | 15-25 | | 16-27 | | | 13-17 | 13-19 | 11-15 |
| n (censored) | | | | 29 | | 11 | | 23 | | 14 | | 18 | | | 6 | 16 | 5 |
| p | | | 0.005 | | | | 0.226 | | | | 0.007 | | | | 0.013 | |  |

Time points: consecutive trials 1, 2, 3, and 4; the median of the ‘time to successful ventilation’ for each technique: MB (Macintosh blade), Bonfils (Bonfils intubation fibrescope), CI (confidence interval), and n (censored): the number of unsuccessful attempts and those taking longer than 90 sec; p: Wald p-values from the stratified Cox model testing for the difference between the techniques in each trial. (missing value (n=1))
