# Supplementary material for: Bonfils intubation fibrescope: use in simulation-based intubation training for medical students in comparison to MacIntosh laryngoscope
Source: BMC Res Notes. 2016 Feb 27;9:127. doi: 10.1186/s13104-016-1937-2 (PMC4769496; doi:10.1186/s13104-016-1937-2)
Supplement: Supplementary file 2 — 10.1186/s13104-016-1937-2 ‘Success probabilities’ of the female and male participants. [file 13104_2016_1937_MOESM2_ESM.docx]

Additional file 2. ‘Success probabilities’ of the female and male participants

| MB trial | | 1 | | 2 | | 3 | | 4 | |
| --- | --- | --- | --- | --- | --- | --- | --- | --- | --- |
|  | gender | | | | | | | | |
| success |  | female | male | female | male | female | male | female | male |
|  | yes | 74  (95%) | 65  (92%) | 77  (99%) | 71  100% | 76  (97%) | 69  (97%) | 73  (94%) | 68  (96%) |
|  | no | 4  (5%) | 6  (9%) | 1  (1%) | 0  (0%) | 2  (3%) | 2  (3%) | 5  (6%) | 3  (4%) |
|  | p | 0.315 | | 0.523 | | 0.654 | | 0.413 | |

| Bonfils trial | | 1 | | 2 | | 3 | | 4 | |
| --- | --- | --- | --- | --- | --- | --- | --- | --- | --- |
|  | gender | | | | | | | | |
| success | success | female | male | female | male | female | male | female | male |
|  | yes | 50  (63%) | 60  (85%) | 56  (71%) | 57  (80%) | 61  (77%) | 65  (92%) | 63  (80%) | 66  (93%) |
|  | no | 29  (37%) | 11  (16%) | 23  (29%) | 14  (20%) | 18  (23%) | 6  (9%) | 16  (20%) | 5  (7%) |
|  | p | 0.003 | | 0.126 | | 0.014 | | 0.017 | |

Time points: consecutive trials 1, 2, 3, and 4; technique: MB (Macintosh blade), Bonfils (Bonfils intubation fibrescope), the numbers (%) of participants with successful (yes) and unsuccessful (no) intubation attempts for each trial; the p-values (McNemar test) for comparison of the success rates between the techniques in each trial. (missing value (n=1))
